# Evaluation of a non‐metallic dual‐port expander for intensity modulated proton therapy

**DOI:** 10.1002/acm2.14512

**Published:** 2024-09-23

**Authors:** Kristen McConnell, Zachary Fellows, James Kraus, Mauricio Acosta, Joseph Panoff, Eduardo Pons, Alonso Gutierrez, Andrew Wroe

**Affiliations:** ^1^ Department of Radiation Oncology Miami Cancer Institute Baptist Health South Florida Miami Florida USA; ^2^ Department of Radiation Oncology Herbert Wertheim College of Medicine Florida International University Miami Florida USA

**Keywords:** breast tissue expander, dual‐port tissue expander, proton therapy

## Abstract

**Purpose:**

To provide a methodology for characterization of the technical properties of a newly developed non‐metallic tissue expander for intensity modulated proton therapy.

**Methods:**

Three tissue expanders (AlloX2‐Pro: plastic‐dual port, AlloX2: metal‐dual port, and Dermaspan: metal‐single port) were deconstructed, CT‐scanned, and modeled in RayStation12A. A 165 MeV single spot was used to create RayStation dose planes, and the integrated depth dose profiles were calculated and the DR90 extracted to predict water equivalent thickness (WET). These predictions were compared to measurements taken with an IBA Giraffe MLIC. Native, water, and fully modelled overrides were compared for the AlloX2 Pro to quantify differences in override choices. Geometric considerations between expanders were compared using a ray‐tracing technique to contour the “no‐fly” zone around metallic components using a clinical, three beam arrangement. Lastly, a planning and evaluation framework was provided using a single plan as an illustration.

**Results:**

The measured AlloX2‐Pro WET values were within 0.22 cm of RayStation predictions while metallic values ranged from 0.08 to 0.46 cm. Using natively scanned density values for the AlloX2 Pro improved the discrepancy in WET between predicted and measured from −0.22 to −0.09 cm (drain) and from −0.17 to −0.12 cm (injection). The “no‐fly” zone volume of all three beams reduced 95% between the AlloX2‐Pro and Dermaspan, which geometrically allowed more uniform coverage behind the port and reduced need for beam modulation.

**Conclusion:**

The beam perturbation of the AlloX2‐Pro is well modeled, but improved agreement with measured WET values was observed when utilizing native densities in calculations. The AlloX2 Pro can support beam arrangements that traverse the ports, which can enable simpler beam geometry and a reduction in dose modulation around the port to promote improved robustness and treatment delivery quality.

## 1 INTRODUCTION

Breast cancer accounts for 30% of all female cancers.^1^ Mastectomy is widely utilized with 75% of plastic surgeons in the United States using a two‐stage tissue expander/implant reconstructive approach.^2^ There are two general types of expanders: single and dual port. A single port offers direct access to the expander while the dual port allows additional access to the surgical cavity for periprosthetic aspiration. In an analysis of 1605 prosthetic breast reconstructions and 48 seromas, complication rates were significantly higher for patients with seroma. In this series, 18.8% of seroma patients suffered infection and seven of those nine required explantation.^3^ A study by Parmeshwar et al. compared post operative complications between single and dual port expanders. While they found no statistically significant differences, they did observe that dual‐port expanders significantly reduced postoperative ultrasound imaging as well as delayed IR drain placement.^4^ Frank et al. reported on their experience using dual‐port expanders in the pre‐pectoral space without acellular dermal matrix or other supportive material. This method prevents the placement of a closed suction drain, and they reported that the dual‐port expander offers a safe alternative that might decrease the patient's pain and discomfort that is often associated with the closed suction drains.^5^

Adjuvant radiation therapy is required in some patients to ensure significant local control and long‐term survival. Many advances in the breast cancer treatment paradigm have led to long‐term disease control, thus leading to an emphasis on preventing iatrogenic radiation induced morbidity. When radiation is delivered to breast cancer patients, the heart and lungs are important to protect from unnecessary radiation. Radiation‐associated cardiac effects manifest as acute or late injuries. Acute effects include pericarditis and late include congestive heart failure, ischemia, coronary artery disease, or myocardial infarction several months to years after radiation.^6^ In addition to concerns about mean heart dose, toxicity from radiation of endothelial lining of blood vessels in specific arteries is a concern.^7^, ^8^ Radiation of arteries may cause progressive functional changes like pro‐inflammatory responses.^8^ A recent systematic review of ionizing radiation and cardiovascular disease showed a causal association between radiation and cardiovascular disease at both high and low doses.^9^ Proton therapy is known to reduce normal tissue integral dose and many studies have shown the dosimetric superiority of protons for mean heart dose when compared with photons.^7^, ^10^, ^11^, ^12^, ^13^ Clinical trials are also underway including the RADCOMP trial (NCT02603341) to compare cardiac events in protons versus photons in the scenario of nonmetastatic breast cancer with comprehensive nodal radiation (including internal mammary nodes).^10^

In the cases of patients with tissue expanders who require adjuvant radiation, both photons and protons have been used. The traditional approach for photons requires the use of steep tangents which results in higher lung and heart doses, particularly when covering regional lymph nodes.^14^, ^15^ The technical challenge is largely geometric because the tangents must be steep to maintain coverage without increasing the contralateral breast dose. This often results in a higher heart and lung dose. Using protons, this geometric challenge is eliminated by using en face anterior oblique beams delivered in a spot scanned delivery. However, for patients with tissue expanders, proton delivery using intensity modulated proton therapy (IMPT) is still challenging. The tissue expanders are generally filled with saline but have a metal encased port with a magnet located on the front of the expander. This metal is a cause for concern in terms of range uncertainty of the proton beam.^16^ Treatment techniques exist to avoid beams going through the expander port, thereby reducing the uncertainty in delivery.^15^, ^17^, ^18^ These studies are all in the setting of a single‐port tissue expander. Provided the separation from the distal edge of the port to the chest wall is sufficiently large, the three‐beam arrangements described in the literature can achieve prescription dose at the chest wall behind the expander port. In the case of dual port expanders, selecting beams that avoid traversing the metal ports, maintain prescription coverage behind the ports, and do not traverse the contralateral breast becomes challenging. Despite the benefits in the reconstructive process with dual‐port expanders, women receiving proton radiation traditionally need single port expanders with dual‐port expanders generally being a contraindication for protons at many clinics.

Sientra has developed the AlloX2 Pro, a dual‐port expander that utilizes plastic ports and a small magnet centered on the device. The use of plastic material in the expander ports may reduce range uncertainties associated with metal ports and allow beams to traverse the ports. This could enable the use of dual‐port expanders with IMPT while still reducing the uncertainties associated with traditional metal single and dual‐port expanders. Given the newness of this device to the clinical environment, it is important for each institution to establish whether this form of dual port expander is suitable for proton therapy at the respective center, including the appropriateness of the institution's current planning and delivery techniques. This manuscript does not include a robust planning study to evaluate planning techniques, but rather provides a methodology for characterization of the technical properties of the expander for institutions looking to use this new device clinically. The scope of this work included building a digital model of the AlloX2 Pro and two clinically available metallic single/dual port expanders from Sientra, comparing the predicted versus measured water equivalent thickness (WET), evaluating the impact of using overrides on the AlloX2 Pro, identifying geometric differences with potential impact on planning, and establishing a framework for a planning study for the AlloX2 Pro.

## 2 METHODS

**FIGURE 1:**
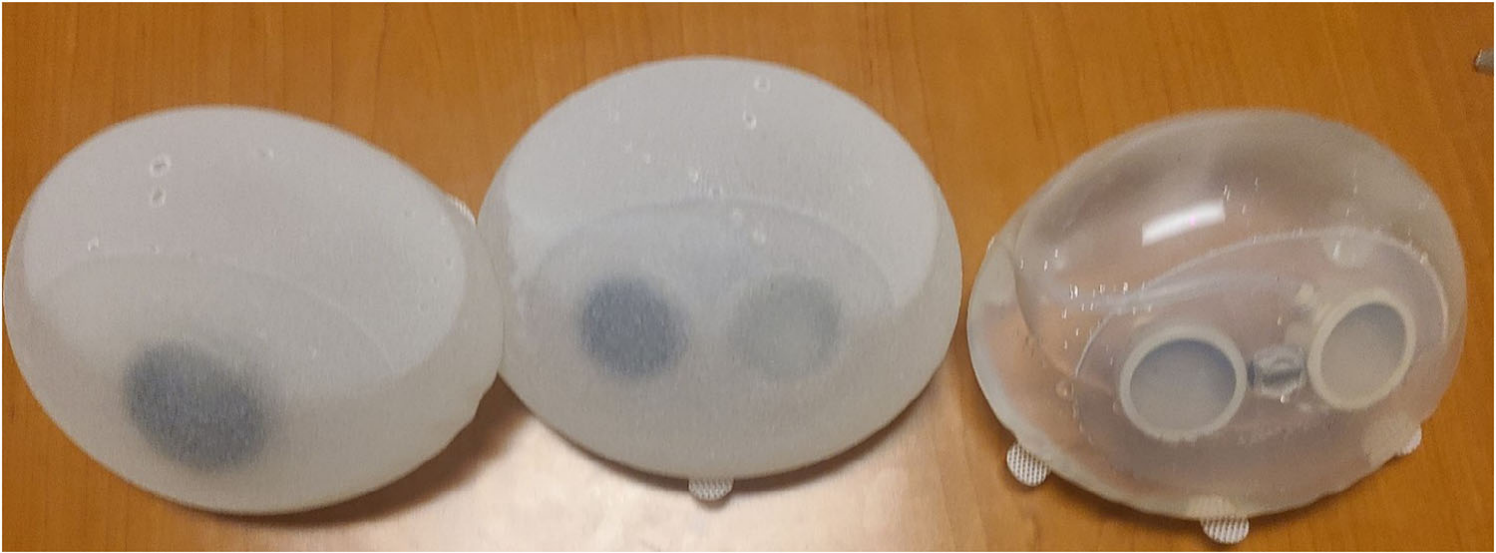
Fully filled expanders: Dermaspan (left), AlloX2 (middle), and AlloX2 Pro (right).

The three expanders used in this study were the Dermaspan (single metal port), AlloX2 (dual metal port), and AlloX2 Pro (dual plastic port). Figure 1 shows these three expanders filled with the same amount of saline. The three types of expanders all utilize a similar design by having a silastomer (polydimethylsiloxane with silicate stabilizer) bag filled with saline that contains one or two drain or injection ports. External access of these ports is achieved using an embedded high‐density magnet, either encased in the port or the silastomer bag. The AlloX2 Pro makes use of a smaller magnet located in between the plastic injection/drain ports, while the other two models utilize a magnet directly embedded into the metallic casing of the injection/drain ports.

### 2.1 Imaging

A CT scan of each expander model was collected using our Siemens Somatom scanner at a 3 mm slice thickness, 500 cm field of view, and 120 kVp/180 mAs. For the scans, each expander was filled with the same amount of saline and scanned on a 10 cm block of solid water. Next, each expander type was deconstructed down to the ports and placed flat on 20 cm of solid water. These were scanned using the same protocol as the filled expanders but with 1 mm slice thickness. Lastly, a 20 cm block of solid water without the expander ports was scanned. A 3 mm slice thickness was selected for the intact expanders to match the institution's clinical protocol, while a 1 mm slice thickness was selected for the deconstructed ones to obtain accuracy for modeling. The extremity IMAR filter was applied to all scans to reduce streaking and photon starvation artifact in all scans, which is our strongest metal artifact reduction reconstruction algorithm. These images were imported into our clinical RayStation 12A install.

### 2.2 Deconstruction and modeling

Measurements of the deconstructed expanders were taken using calibrated calipers and compared against vendor‐provided data. The measurements were used with the ROI geometry tools to create a 3‐D model inside RayStation. A material was assigned to each component of the model as appropriate for the Monte Carlo dose calculation. RayStation 12A allows the creation of any material by specifying the elements, elemental weight, and mean excitation energy. For components that utilized water, air, or titanium, the identical material in RayStation was selected. For other materials not offered in RayStation, materials were created out of their constituent elements. New materials were made for silastomer, neodymium, and PEEK. Neodymium was modeled as a pure element rather than the alloy, Neodymium 35EH. Table 1 shows the materials assigned during modeling for each of the expander components.

**TABLE 1:** RayStation material modeling parameters.

	Perfect model			Chosen RayStation 12A model		
**Water**	H_2_O	Atomic number	[1, 8]	“Water”	*Same as ideal*	
		Elemental weights	[0.112, 0.888]			
		Density	1.00 g/cc			
		Mean excitation energy	75 eV			
**Air**	n/a	Atomic number	[7, 8, 18]	“Air”	*Same as ideal* *(∼99% made up of N_2,_ O_2,_ and Ar)*	
		Elemental weights	[0.755, 0.232, 0.013]			
		Density	0.001 g.cc			
		Mean excitation energy	85.70 eV			
**Polydimethylsiloxane** (with silicate stabilizer)	CH_3_[Si(CH_3_)_2_O]_n_ Si(CH_3_)_3_	Atomic number	[1, 6, 8, 14)	“Water”	Atomic number	[1, 8]
		Elemental weights	Depends on repeating monomer units		Elemental weights	[0.112, 0.888]
		Density	0.965 g/cc		Density	1.00 g/cc
		Mean excitation energy	Depends on repeating monomer units		Mean Excitation Energy	75 eV
**Neodymium 35EH**	Nd_2_Fe_14_B	Atomic number	[60, 26, 5]	“Nd”	Atomic number	[60]
		Elemental weights	[0.267, 0.723, 0.01]		Elemental weights	[1]
		Density	7.4 g/cc		Density	7.0 g/cc
		Mean excitation energy	546–580 eV^a^		Mean Excitation Energy	546 eV^a^
**PEEK**	C_19_H_14_O_3_	Atomic number	[1, 6, 8]	“PEEK”	Atomic number	[1, 6, 8]
		Elemental weights	[0.048, 0.786, 0.166]		Elemental weights	[0.048, 0.786, 0.166]
		Density	1.323 g/cc		Density	1.323 g/cc
		Mean excitation energy	74 eV^a^		Mean Excitation Energy	74 eV^a^
**Titanium (Grade 2)**	Ti^b^	Atomic number	[22]	“Titanium”	*Same as ideal*	
		Elemental weights	[1]			
		Density	4.54 g/cc			
		Mean excitation energy	233.00 eV			

^a^ These values were estimated using the quantum mechanical approach.

^b^ Per the American Society for Testing and Materials (ASTM) standards for Titanium (Grade 2), the maximum iron, oxygen, carbon, nitrogen, and hydrogen maximum percentage is 0.3%, 0.25%, 0.08%, 0.03%, and 0.015%, respectively. Because of this, the titanium components are well modeled as pure titanium.

### 2.3 WET prediction and evaluation

For the WET prediction and measurement, the correct energy needed to be selected to ensure that no fluence would pass outside of the diameter of the expander components. To check this, the institution's beams’ 6σ values per energy were investigated. The 165 MeV beam has a 6σ of 2.256 cm, which was smaller than the diameters of all the expander ports.

The empty scan with a 20 cm water block was used to create a single spot plan of 165 MeV. All solid water was overridden to water. The models with their respective overrides were overlaid on the scans of the deconstructed ports. Single spots were directed through the center of each of the Dermaspan single port, AlloX2 drain/injection ports, and AlloX2 pro drain/injection ports. The RT DICOM dose files were exported and the dose on each plane was integrated to create an integrated depth dose (IDD) profile for each spot through its respective port. These data were post‐processed to find the distal range 90 (DR90) values for each plan. The DR90 values for each port were subtracted from the DR90 values for the empty plan to calculate the predicted range pullback, or WET.

The IBA Giraffe Multi‐Layer Ion Chamber (MLIC) detector uses a stack of 180 independent vented parallel plate ion chambers to measure range. Each chamber has a 12 cm diameter collecting electrode and a 0.1 cm air gap between chambers. The manufacturer quoted range accuracy for the Giraffe detector is ±0.5 mm.^19^ Using the past annual DR90 range measurements at this institution, the DR90 measured in water was within ±0.3 mm of the Giraffe's measured DR90, which is in line with the Baumer et al. findings.^20^ A set of baseline range measurements were taken using a single spot of 165 MeV with the thin plastic window left on the device. Next, the expander components were individually taped to the front of the thin plastic window and the center of each component was aligned to the lasers. The 165 MeV spot was delivered to each component three times, with re‐setup in between. The RayStation predicted WET values were then compared against the measured WET values and the difference between the predicted/measured reported.

### 2.4 Quantification of override impact on AlloX2 Pro modeling

Two scenarios for the overrides for the AlloX2 Pro were evaluated: (1) the modeled AlloX2 Pro's PEEK overrides were changed to water, and (2) the model was removed from the AlloX2 Pro and native values from the CT calibration curve were used. When changing to a water override all other material overrides (air and silastomer) were left alone, and when using the native density, the model was removed completely for all overrides. A 165 MeV spot was directed through each port in the same way as above and the IDD was used to calculate the distal R90 and subsequent predicted WET. This range pullback was compared to the WET value calculated from the overridden PEEK AlloX2 Pro to understand the impact of the model.

### 2.5 Geometric considerations

In addition, the geometrical considerations for planning were compared using a three‐beam clinical arrangement for all three expander types and using a ray tracing method to project the shadow of avoidance region from the beam's eye view. This shadow of the avoidance regions is the overlapping region of all three beams where none of the three beams can put spots on target. For the Dermaspan and the AlloX2, the avoidance region was the metallic ports plus 0.5 cm, and for the AlloX2 Pro, it was the small magnet plus 0.5 cm. The three beams used were our typical clinical arrangement: (Beam 1) RAO 60°, (Beam 2) AP beam, and (Beam 3) LAO 50°. These beams were applied the same for each expander type. The ray trace structure was created for each beam, then algebra was performed to visually show the so called “no‐fly” zones for each beam, which is an important aspect of understanding expander planning when using an avoidance region. The volume was reported for the “no fly” zone where none of the beams could access and compared across the three types.

### 2.6 Suggested approaches for evaluating clinical overrides

Given the differences in material and shape of avoidance regions between the commonly used metallic expanders and the AlloX2 Pro, it is critical to establish the clinical impact of these differences through a planning study before implementation into clinic. Here, a single replanned case is shown to demonstrate a framework to evaluate the impact. This method can be employed as a part of a larger institution specific planning study when evaluating for use in clinic. Following institutional guidance, a patient's scan who was previously treated with a metallic expander was used to insert an AlloX2 Pro 3D Model in RayStation. The AlloX2 Pro 3D model had material overrides, and the region outside the AlloX2 Pro model and inside the saline implant was overridden to water. A clinically acceptable left breast and regional nodes plan to 50.4 GyRBE utilizing a three‐beam approach was created. The three beams were the institution's typical set for a left sided expander case: RAO at 350°, an LAO at 20°, and an LAO at 50°. The beams were directed through the plastic ports but avoided the small neodymium magnet in the center with a 0.5 cm avoidance margin per standard. The plan was optimized using a 0.5 cm setup uncertainty and 3.5% range uncertainty on a 3 mm dose grid as per the institution's clinical standard. The plan was evaluated as if it were a clinical plan, and it met all the nominal and robust criteria as well as successfully prevented spots inside the 5 mm magnetic avoidance region. To demonstrate the method for evaluating the clinical impact of overrides for the AlloX2 Pro, the plan was recalculated as an evaluation dose (“fixed MU”) with the port components made from PEEK overridden to water. The converse was also performed by optimizing as if the entire expander (except the magnet and drain air pocket) was water. Then the plan was recalculated an as evaluation dose with the proper overrides in place for PEEK. Institutional coverage and OAR sparing metrics should be evaluated to understand how differences in override strategy affect the clinical plan metrics. The institutional coverage requirement for the chest wall, supraclavicular nodes, axillary nodes, and internal mammary nodes is 97% of the volume is covered by 95% (47.88 Gy). These values as well as the mean heart dose were reported and compared for each plan.

## 3 RESULTS

### 3.1 Imaging

**FIGURE 2:**
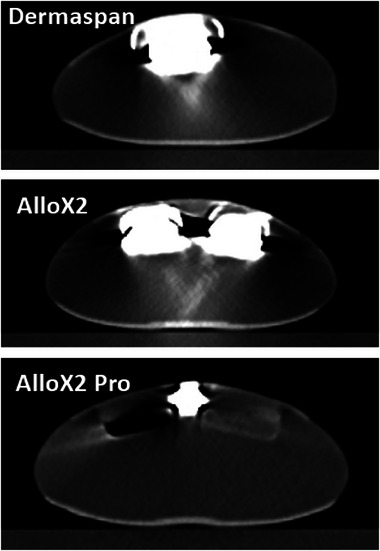
Comparison of iMAR CT scans of Dermaspan (top), AlloX2 (middle), and AlloX2 Pro (bottom).

Figure 2 shows the three IMAR scans of the fully filled expanders. From top to bottom in the figure, the Dermaspan with the single metal port, the AlloX2 with the dual metal ports, and the AlloX2 Pro with the dual plastic ports are shown. Visual differences in the scans are identified, specifically when observing differences in artifact. The small magnet in the AlloX2 Pro creates a small streaking artifact but is smaller in footprint than the streaking artifacts seen in the Dermaspan and AlloX2. There are also significant photon starvation artifacts around the ports on the Dermaspan and AlloX2, which does not exist around the ports on the AlloX2 Pro. There is still some photon starvation artifact on the AlloX2 Pro around the small magnet.

### 3.2 Deconstruction and modeling

**FIGURE 3:**
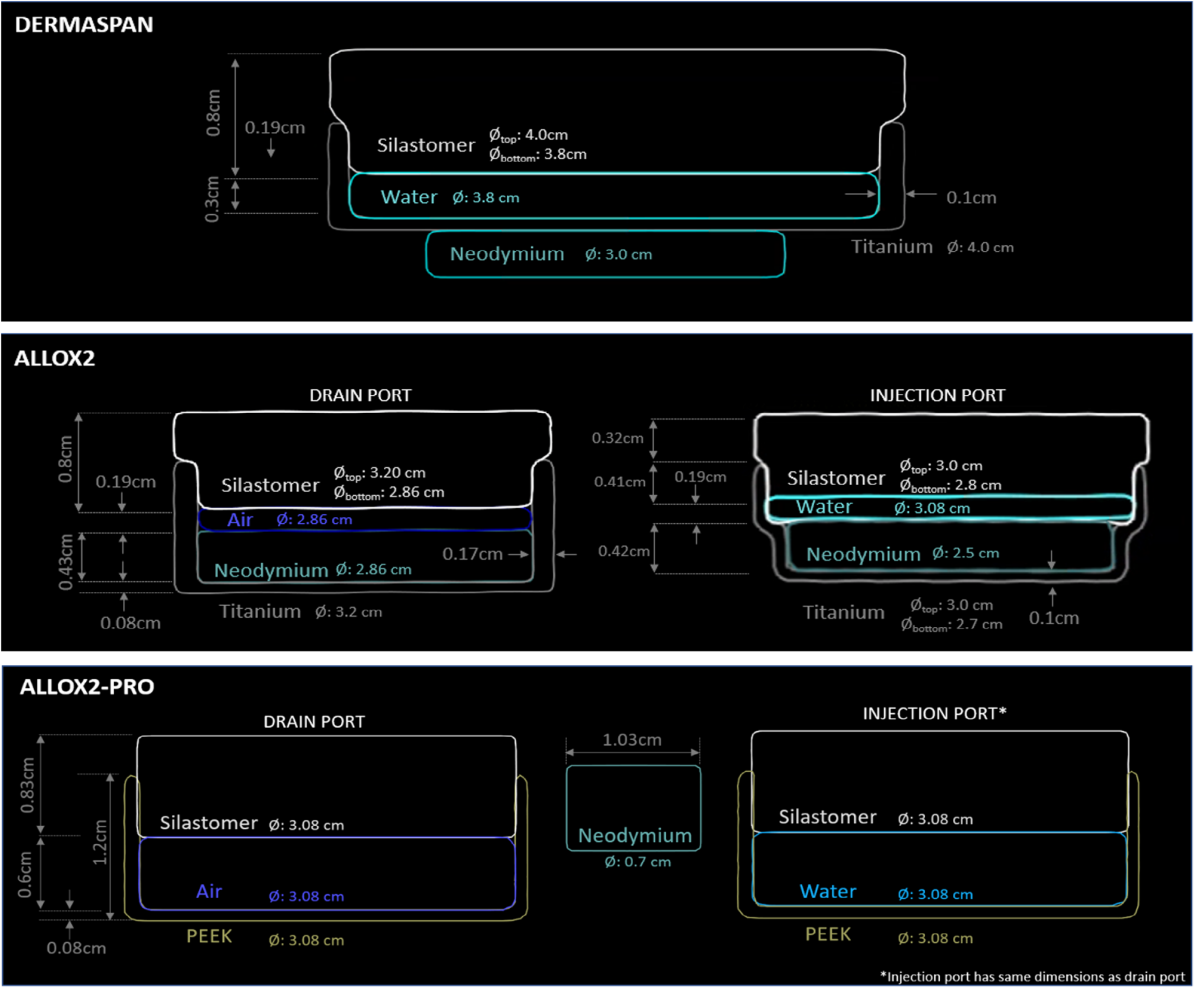
Drain/injection port modeling dimensions for the Dermaspan (bottom), AlloX2 (middle), and AlloX2 Pro (bottom).

Figure 3 shows the results of measuring each component of each deconstructed expander. The physical ports are press fit onto the silastomer expander, thus they were modeled with the silastomer inside of the PEEK or titanium port casing structure. For the Dermaspan, the magnet is outside titanium casing while, for the AlloX2, the magnet is housed inside the titanium casing. For all expanders, the magnets were modeled as pure neodymium.

**FIGURE 4:**
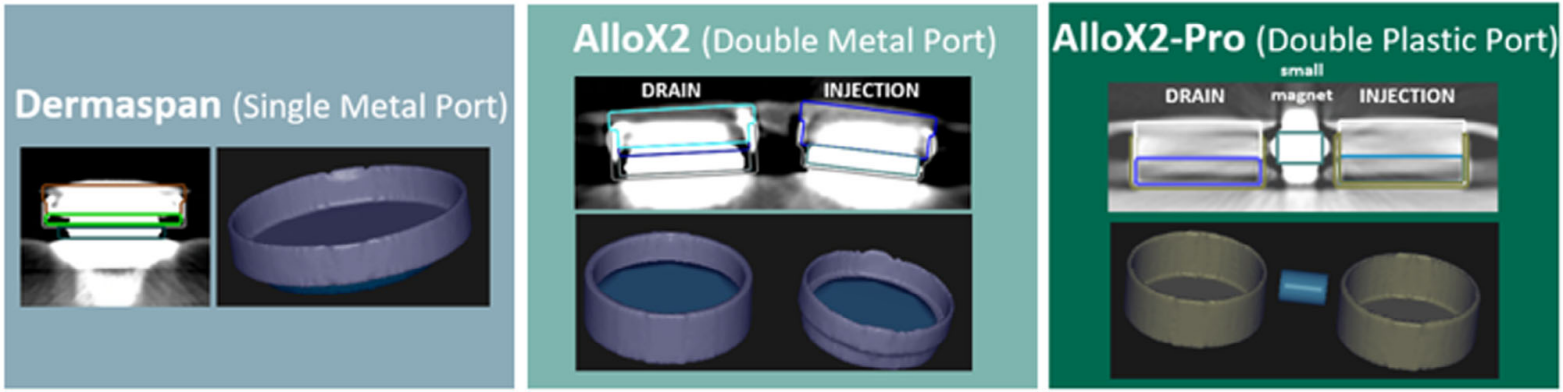
The RayStation template models for the Dermaspan (left), AlloX2 (middle), and AlloX2 Pro (right).

Figure 4 shows the final model templates created in RayStation overlaid with a CT scan of the internal parts. These can be imported and moved independently to match the curvature of the expander once inside the body for clinical patients.

### 3.3 WET prediction and evaluation

Table 2 shows the physical thickness, the distal range 90% value, the RayStation 12A predicted WET, the measured WET, and the discrepancy and percentage difference between predicted and measured for each expander type. For the AlloX2 and the AlloX2 Pro, part of the drain port was filled with air during the simulation and the measurement. This is because the drain port directly accesses the chest wall cavity and is not filled with saline from the tissue expander. The discrepancies and absolute percentage differences between predicted and measured WET were −0.08 cm/3.24%, 0.36 cm/13.58%, 0.46 cm/16.73%, −0.22 cm/21.57%, and −0.17 cm/10.73% for the Dermaspan drain, AlloX2 drain, AlloX2 injection, AlloX2 Pro drain, and AlloX2 Pro injection ports, respectively. The largest discrepancy between the predictions and the measurements is observed in the case of the AlloX2 metal injection port (overestimated by 0.46 cm).

**TABLE 2:** Physical thickness, predicted WET, and measured WET values.

		Physical (cm) Includes proximal silastomer	Predicted DR 90 (cm)	Predicted WET (cm)	Measured WET (cm)	Discrepancy (cm)	Absolute percentage difference
**Water only**			18.59				
**Dermaspan**	Drain	1.17	16.17	2.43	2.51	−0.08	3.24%
**AlloX2**	Drain	1.5	15.77	2.83	2.47	0.36	13.58%
	Injection^a^	1.34	15.62	2.98	2.52	0.46	16.73%
**AlloX2 Pro**	Drain	1.51	17.68	Modeled: 0.91	1.13	−0.22	21.57%
			17.70	Water: 0.89		−0.24	23.76%
			17.55	Native density: 1.04		−0.09	8.29%
	Injection^a^	1.51	17.09	Modeled: 1.50	1.67	−0.17	10.73%
			17.11	Water: 1.48		−0.19	12.06%
			17.04	Native density: 1.55		−0.12	7.45%

^a^ Filled with liquid during the irradiation.

### 3.4 Quantification of override impact on AlloX2 Pro

Table 2 also includes the WET results of using water and the native scanned density (no model) when predicting the WET. For the AlloX2 Pro drain port, the discrepancies and absolute percentage differences between predicted and measured were −0.22 cm/21.57%, −0.24 cm/23.76%, and −0.09 cm/8.29% for modeling as PEEK, water, and native density (no modeling), respectively. For the AlloX2 Pro injection port, the discrepancies and percentage differences between predicted and measured were −0.17 cm/10.73%, −0.19 cm/12.06%, and −0.12 cm/7.45% for modeling as PEEK, water, and native density (no modeling), respectively. The native density showed the least discrepancy and percentage difference for the AlloX2 Pro's predicted versus measured WET.

### 3.5 Geometric considerations

**FIGURE 5:**
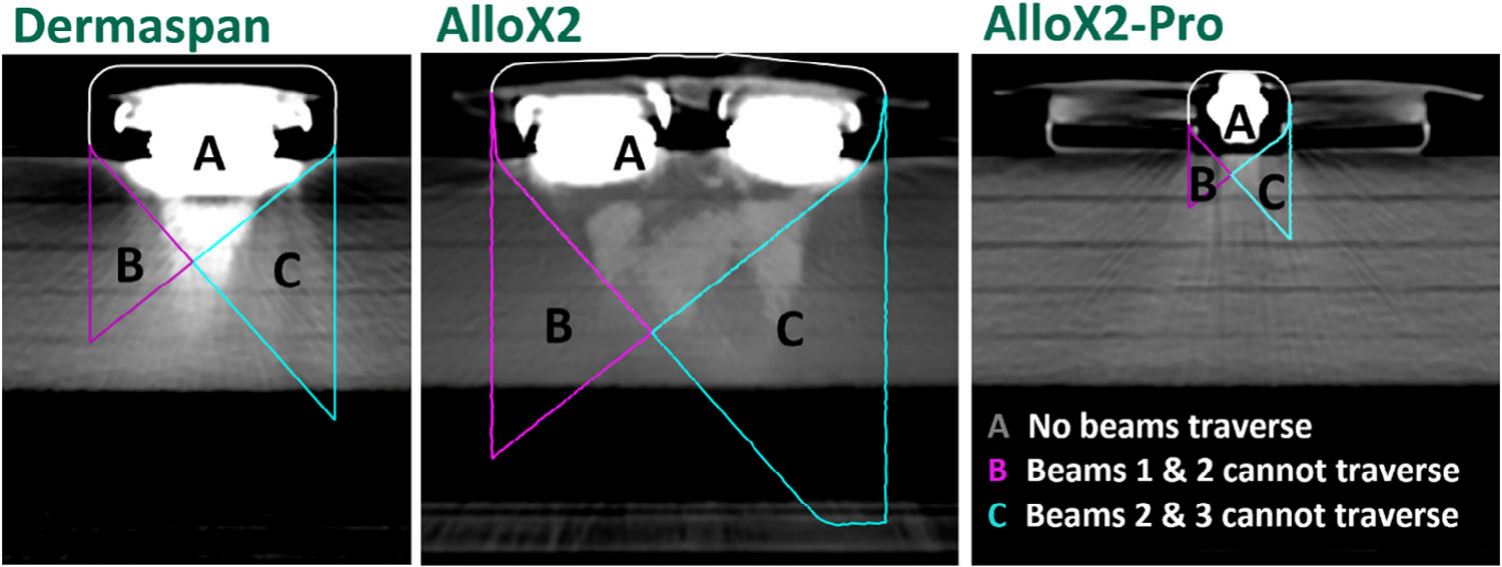
“Nofly” zones of the three different expanders as calculated using a ray trace method.

The calculated “no fly” zones for each expander type are shown in Figure 5. The zones are partitioned into A, B, and C. Portion A is representative of the region where no beams traverse, portion B is representative of where beams 1 (RAO) and 2 (AP) cannot traverse, and portion C is representative of where beams 2 (AP) and 3 (LAO) cannot traverse. The volumes for portion A were 53.47 cc, 104.58 cc, and 4.30 cc for the Dermaspan, AlloX2, and AlloX2 Pro, respectively. There was a 95% reduction in the size of the portion A “no fly” zone when using the AlloX2 Pro.

### 3.6 Suggested approaches for evaluating clinical overrides

Table 3 shows the clinical reported metrics for targets and the heart for the differently optimized plans using the AlloX2 Pro. The impact on clinical metrics whether the plastic ports are modeled as their respective component material, PEEK, or modeled as water during the optimization showed less than 1% reduction in target coverage when recalculated on the opposite override. For the OAR sparing, the effect on average heart dose was at most an increase of 0.02 Gy, and the effect on lung V20 was an increase in 0.28%

**TABLE 3:** Clinical reported metrics for targets and the heart for the differently optimized plans using the AlloX2 Pro.

		Plan optimized with PEEK override on plastic components (all other overrides as modeled)		Plan optimized with Water override on plastic components (all other overrides as modeled)	
Target/OAR	Metric	Nominal	Recalculated on water	Nominal	Recalculated on PEEK
CTV_CW_L_Eval	At least volume 97% at 47.88 Gy	98.88%	98.88%	98.33%	98.31%
CTV_AX_L	At least volume 97% at 47.88 Gy	100.00%	99.98%	99.93%	99.93%
CTV_SC_L	At least volume 97% at 47.88 Gy	99.96%	99.96%	99.93%	99.98%
CTV_IMN_L	At least volume 97% at 47.88 Gy	100.00%	100.00%	100.00%	99.98%
Heart	At most 2 Gy (RBE) average dose	1.70 Gy (RBE)	1.72 Gy (RBE)	2.09 Gy (RBE)	2.09 Gy (RBE)
Lung	V20_Gy_	13.34%	13.62%	14.47%	14.30%

## 4 DISCUSSION

The presence of artifact and starvation regions on all three scans likely necessitates the need for overrides of some kind for IMPT planning. Overriding the starvation or artifact regions may be necessary for accurate dose calculations depending on the beam angles selected. In the AlloX2 Pro, the reduction in streaking artifact could allow users to reduce how much artifact is overridden as the overrides appear limited to the region of the small magnet.

The WET values in Table 2 show that the AlloX2 Pro plastic components can be reasonably modeled in RayStation12A within 0.22 cm for the drain port and 0.17 cm for the injection port; however, the drain port can be modeled within 0.09 cm and the injection port within 0.12 cm when simply using the native scanned density values Many centers are used to overriding the expander components to the correct densities, but for the AlloX2 Pro, the results of the WET testing suggests that is not needed for these plastic expanders. For the Dermaspan and AlloX2, the presence of titanium and neodymium in the ports results in a modeling versus measurement discrepancy on the order of 0.1–0.46 cm. This is consistent with the field's standard practice to avoid shooting through these components in an effort to reduce uncertainty.^15^, ^16^, ^17^, ^18^

**FIGURE 6:**
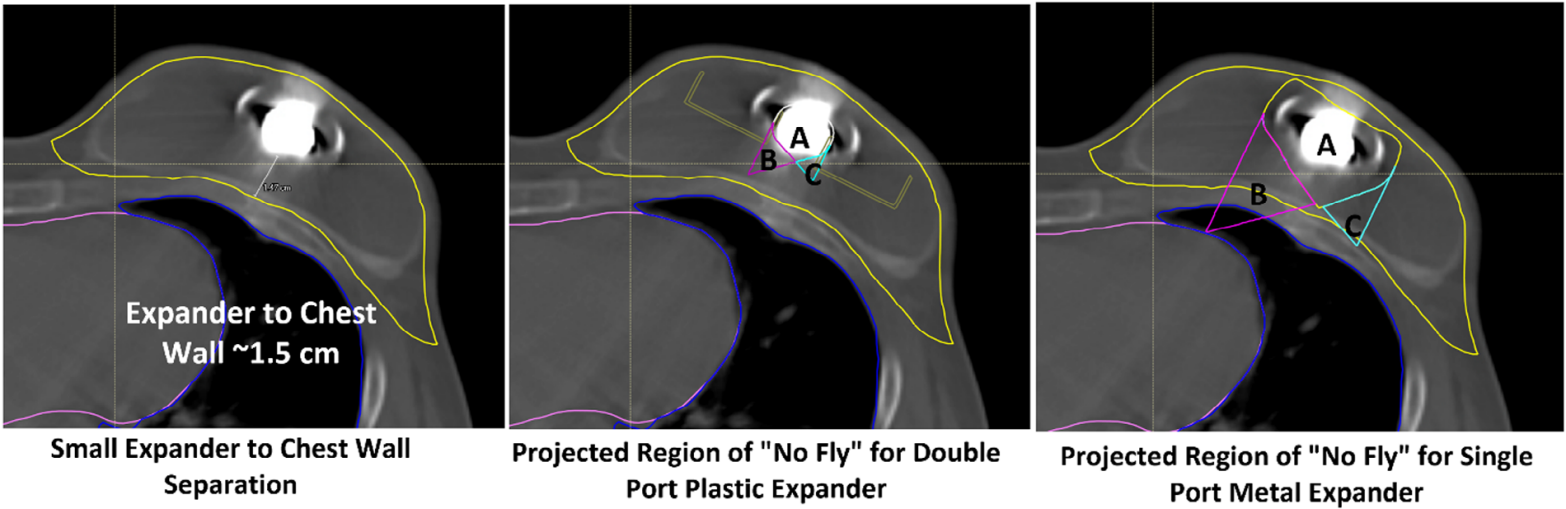
No fly zones overlaid on clinical breast plan.

The reduction in absolute percentage differences between modeled and predicted WET between the dual port metallic expander and the AlloX2 Pro could enable the use of the dual port expander in protons. Furthermore, it could reduce the uncertainty seen with directing beams through the ports. The plastic AlloX2 Pro ports could allow planners to traverse the plastic ports with a clinically acceptable uncertainty. This would reduce the geometric challenges that dual port metal expanders face by dramatically reducing the size of the avoidance region and consequently reducing the so called “no fly zone” that was observed with the 95% reduction in “no fly” zone using the AlloX2 Pro with an avoidance of only the magnet plus 5 mm. For those clinics observing the rule that every portion of the target must be treated with two beams, this smaller avoidance region has clinical advantages. Figure 6 shows a case where the expander was not filled to its maximum and had a separation from the port to chest wall of about 1.5 cm. With a single port metal expander, there were portions of the chest wall where only one beam could successfully deliver dose. If the patient had a dual plastic expander, the “no fly” zones fit entirely inside the implant, allowing for two beams to treat the chest wall.

Additionally, an important factor in improved uncertainty is not just how well predicted versus modeled matches, but how closely the port WET is from the material WET around it as dramatic changes in WET can have downstream effects if the port is in a different place for treatment delivery. This can be observed by comparing the measured WET and the physical thickness of the ports. For the AlloX2, the physical thickness of the drain and injection ports were 1.50 and 1.34 cm, while the measured WET was 2.47 and 2.52 cm. For the AlloX2 Pro, the physical thickness of the drain and injection ports were 1.51 cm for both, while the measured WET was 1.13 cm for the drain and 1.67 for the injection. If the beam traversed the port like it were perfect water, the measured WET and physical thickness would be equal values. How much these differ could have downstream impacts on the plan quality if the expander ports move around. There is a larger difference in the AlloX2, which means the placement of the ports relative to the beam at treatment will have a different downstream impact than the AlloX2 Pro with its smaller difference. The AlloX2 Pro may allow for clinically acceptable uncertainty that may not require as stringent setup guidelines, which needs assessed in a planning study.

Currently at our institution, during treatment delivery of breast expander cases, the therapists are instructed to use orthogonal kV and cone beam CT to ensure the expander ports are inside an image guidance avoidance structure. There are daily variations in the setup of these cases as patient anatomy may change and the ports potentially move relative to this change. The use of the AlloX2 Pro could evolve the setup and proton delivery workflow leading to less focus on port placement relative to patient anatomy since therapists would only need to match the small magnet to the image guidance structure.

The framework outlined for clinically evaluating the differences of the models for the AlloX2 Pro includes replanning cases with the AlloX2 in place with modeled overrides and recalculating with a water override and native densities. For the single plan showed to demonstrate the methodology, there were small differences in coverage (less than 1%/less than 1 Gy) when looking at the re‐calculated plans with the overrides/no overrides in place. These clinical metrics can be used to understand the clinical significance of the override choices in your AlloX2 Pro prior to clinical implementation.

## 5 CONCLUSION

The beam perturbation of the AlloX2 Pro can be modeled by RayStation 12A, but better agreement between measured and predicted, for both drain and injection port, was observed when using the native CT scanner densities. Due to the ability to use the native density, utilization of the AlloX2 Pro can support beam arrangements that traverse the plastic ports without material overrides and a smaller beam avoidance region. This is important for IMPT treatment planning because it means less modulation around the port, which will promote improved robustness and treatment delivery quality. Furthermore, the material difference between the AlloX2 Pro ports and the surrounding expander water is lower than that of metal. This is important for reducing uncertainty in treatment delivery as the position of the port at treatment may have less of an impact on plan quality. The next step in evaluating this device for clinical use is a planning study aimed at comparing the planning techniques and dosimetric outcomes using the three expander choices.

## AUTHOR CONTRIBUTIONS

All authors contributed directly to the intellectual content of the paper. Our financial disclosures have been documented in the electronic submission.

## CONFLICT OF INTEREST STATEMENT

This work was done as part of a funded study by Sientra, Inc.
